# Phenogrouping heart failure with preserved or mildly reduced ejection fraction using electronic health record data

**DOI:** 10.1186/s12872-024-03987-9

**Published:** 2024-07-05

**Authors:** Fardad Soltani, David A. Jenkins, Amit Kaura, Joshua Bradley, Nicholas Black, John P. Farrant, Simon G. Williams, Abdulrahim Mulla, Benjamin Glampson, Jim Davies, Dimitri Papadimitriou, Kerrie Woods, Anoop D. Shah, Mark R. Thursz, Bryan Williams, Folkert W. Asselbergs, Erik K. Mayer, Christopher Herbert, Stuart Grant, Nick Curzen, Iain Squire, Thomas Johnson, Kevin O’Gallagher, Ajay M. Shah, Divaka Perera, Rajesh Kharbanda, Riyaz S. Patel, Keith M. Channon, Richard Lee, Niels Peek, Jamil Mayet, Christopher A. Miller

**Affiliations:** 1 Division of Cardiovascular Sciences, School of Medical Sciences, Faculty of Biology, Medicine and Health, Manchester Academic Health Science Centre, University of Manchester, Oxford Road, Manchester, M13 9PL UK; 2 Manchester University NHS Foundation Trust, Southmoor Road, Wythenshawe, Manchester, M23 9LT UK; 3 Division of Informatics, Imaging and Data Sciences, School of Health Sciences, Faculty of Biology, Medicine and Health, University of Manchester, Oxford Road, Manchester, M13 9PL UK; 4 NIHR Imperial Biomedical Research Centre, Imperial College London and Imperial College Healthcare NHS Trust, St Mary’s Hospital, London, W2 1NY UK; 5 Imperial Clinical Analytics, Research and Evaluation, Digital Collaboration Space, Faculty of Medicine, Imperial College London and Paddington Life Sciences, London, UK; 6 NIHR Oxford Biomedical Research Centre, University of Oxford and Oxford University Hospitals NHS Foundation Trust, Oxford, UK; 7 London Biomedical Research Centre, NIHR University College, University College London and University College London Hospitals NHS Foundation Trust, London, UK; 8 NIHR Leeds Clinical Research Facility, Leeds Teaching Hospitals Trust and University of Leeds, Leeds, UK; 9 NIHR Manchester Biomedical Research Centre, Manchester University NHS Foundation Trust, University of Manchester, Manchester, UK; 10 NIHR Southampton Clinical Research Facility and Biomedical Research Centre, Faculty of Medicine, University of Southampton and University Hospital Southampton NHS Foundation Trust, Southampton, UK; 11 NIHR Biomedical Research Centre, Glenfield Hospital, Leicester, LE3 9QP UK; 12 NIHR Bristol Biomedical Research Centre, University of Bristol and University Hospitals Bristol and Weston NHS Foundation Trust, Bristol, UK; 13 King’s College London British Heart Foundation Centre of Excellence and King’s College Hospital NHS Foundation Trust, London, UK; 14 British Heart Foundation Centre of Excellence, School of Cardiovascular Medicine and Sciences, King’s College London, London, UK; 15 NIHR Biomedical Research Centre, The Royal Marsden and Institute of Cancer Research, London, UK; 16 Wellcome Centre for Cell-Matrix Research, Division of Cell-Matrix Biology & Regenerative Medicine, School of Biology, Faculty of Biology, Medicine & Health, Manchester Academic Health Science Centre, University of Manchester, Oxford Road, Manchester, M13 9PT UK; 17 The Healthcare Improvement Studies Institute (THIS Institute), Department of Public Health and Primary Care, University of Cambridge, Cambridge, UK

**Keywords:** Heart failure with preserved or mildly reduced ejection fraction, Machine learning, Electronic health records

## Abstract

**Background:**

Heart failure (HF) with preserved or mildly reduced ejection fraction includes a heterogenous group of patients. Reclassification into distinct phenogroups to enable targeted interventions is a priority. This study aimed to identify distinct phenogroups, and compare phenogroup characteristics and outcomes, from electronic health record data.

**Methods:**

2,187 patients admitted to five UK hospitals with a diagnosis of HF and a left ventricular ejection fraction *≥* 40% were identified from the NIHR Health Informatics Collaborative database. Partition-based, model-based, and density-based machine learning clustering techniques were applied. Cox Proportional Hazards and Fine-Gray competing risks models were used to compare outcomes (all-cause mortality and hospitalisation for HF) across phenogroups.

**Results:**

Three phenogroups were identified: (1) Younger, predominantly female patients with high prevalence of cardiometabolic and coronary disease; (2) More frail patients, with higher rates of lung disease and atrial fibrillation; (3) Patients characterised by systemic inflammation and high rates of diabetes and renal dysfunction. Survival profiles were distinct, with an increasing risk of all-cause mortality from phenogroups 1 to 3 (*p* < 0.001). Phenogroup membership significantly improved survival prediction compared to conventional factors. Phenogroups were not predictive of hospitalisation for HF.

**Conclusions:**

Applying unsupervised machine learning to routinely collected electronic health record data identified phenogroups with distinct clinical characteristics and unique survival profiles.

**Supplementary Information:**

The online version contains supplementary material available at 10.1186/s12872-024-03987-9.

## Introduction

Heart failure (HF) is a global health priority that carries significant societal and economic impacts [1]. The prevalence of HF, currently estimated to be 1–2% of the adult population, is expected to increase by approximately 50% by the year 2030 [2, 3]. Accounting for approximately half of patients with HF, HF with preserved or mildly reduced ejection fraction includes a heterogenous group of patients with wide-ranging pathophysiological mechanisms, multimorbidity, and variable outcomes [4].

Owing in part to a ‘one-size-fits-all’ approach, almost all phase III trials in HF with preserved or mildly reduced ejection fraction have been neutral. Sodium-glucose co-transporter-2 (SGLT-2) inhibitors are associated with reduced risk of hospitalisation for heart failure and improved quality of life; however, no intervention has demonstrated mortality benefit [5, 6]. Reclassification into more distinct phenogroups as a basis for targeted therapies is a priority.

Whilst previous studies applying machine learning techniques to HF datasets suggest subgroups exist, they have generally been limited to data from randomised controlled trials or retrospective cohorts [7–11]. Electronic health record data collected at scale provide large, unselected cohorts that are reflective of clinical practice, with potentially detailed clinical characterisation and outcome data, and as such are attractive for such analyses [12].

The aims of this study were to apply unsupervised machine learning techniques to electronic health record data in order to identify distinct phenogroups and compare phenogroup characteristics and outcomes.

## Methods

### Study population

Routinely collected electronic health record data were made available from the National Institute for Health Research (NIHR) Health Informatics Collaborative database (ClinicalTrials.gov, NCT03507309), the design of which has been described previously [13–15]. Briefly, any patient who underwent measurement of serum troponin at five UK hospitals (Imperial College Healthcare, University College Hospital, Oxford University Hospital, Kings College Hospital, and Guys and St Thomas’ Hospital) between 2010 (2008 for University College Hospital) and 2017 were eligible for database inclusion. A fully de-identified copy of the database was frozen and made available for analysis on 1st April 2017. The study was approved by the London-South East Research Ethics Committee (REC reference: 16/HRA/3327), and patients were not required to provide consent. Patients admitted with a diagnosis of HF, as determined from International Classification of Diseases 10th Revision (ICD-10) discharge codes, and a left ventricular ejection fraction (LVEF) *≥* 40% on echocardiography, were included in the current study. The National Institute for Cardiovascular Outcomes Research (NICOR) ICD-10 definition of HF was used to define HF (Supplemental Table 1), in keeping with the England and Wales National Heart Failure Audit [16].

### Cluster analysis

Patients were assigned to phenogroups using cluster analysis. Detailed data pre-processing and clustering methodology are provided in the Supplemental Methods. Briefly, 47 baseline variables, selected according to clinical practice and literature review, were considered for cluster analysis. These included demographic, clinical, laboratory and echocardiographic data, as well as invasive angiographic and revascularisation data from the index admission. Variables with *≥* 20% missing data were excluded, leaving a total of 42 variables for further analysis (Supplemental Table 2). The proportion of patients with incomplete data was low (424 [19%] of 2187) and the percentage of missing values for each variable ranged from 0 to 14% (Supplemental Fig. 1). Missing values were imputed by random forest imputation using the *missForest* package in R [17].

Three unsupervised machine learning clustering methods were applied: Density-Based Spatial Clustering and Application with NOISE (DBSCAN), a density-based clustering algorithm that does not require pre-specification of the optimal number of clusters and is well suited to finding arbitrary-shaped clusters [18]; Gaussian mixture modelling, a model-based clustering technique that achieves parameter estimation using an expectation-maximisation algorithm and penalisation of model complexity using the Bayesian Information Criterion (BIC) [19]; and k-means clustering, a commonly used partition-based clustering approach that assigns patients to each cluster using Euclidean distance metrics [20]. The *fpc*, *mclust*, and *stats* packages in R were used for each method respectively. The optimal number of clusters in the k-means clustering algorithm was determined using the *NbClust* R package. Cluster stability was evaluated by repeating the clustering algorithms using bootstrapped replicates and calculating the mean Jaccard coefficient for each cluster. Jaccard coefficient values range between 0 and 1, with a value closer to 1 suggesting greater cluster stability. The *clusterboot* function in R was used to repeat the model-based algorithm 100 times, and the k-means clustering algorithm 1000 times (see below regarding the DBSCAN method).

### Comparison of phenogroups

Clinical characteristics of the phenogroups were compared using a Chi-squared or Fisher exact testing for categorical variables, and an ANOVA or Kruskal-Wallis testing for continuous variables.

The primary outcome for the outcome analysis was all-cause mortality. The secondary outcome was hospitalisation for heart failure (HHF) occurring after the index admission. Vital status was ascertained via NHS Digital, which incorporates national death registry information and local notifications. Only HHF episodes occurring at the hospital of index admission were available within the HIC database. Phenogroup-specific survival was estimated by plotting Kaplan-Meier survival curves and compared using log-rank testing. Risk of primary and secondary outcome was compared across phenogroups using unadjusted Cox Proportional Hazards and Fine-Gray competing risks models, respectively. The added prognostic value of phenogrouping was evaluated by assessing model performance over a series of nested (i.e., with or without the phenogroup membership variable) Cox Proportional Hazards models [21]. Specifically, the nested models were compared using the likelihood ratio test and by calculating the C-statistic. Troponin underwent log transformation prior to modelling. The proportional hazards assumption was verified by visualising scaled Schoenfeld residuals. Survival analyses were performed using the *Survival*, *Survminer*, and *cmprsk* R packages.

## Results

**Fig. 1:**
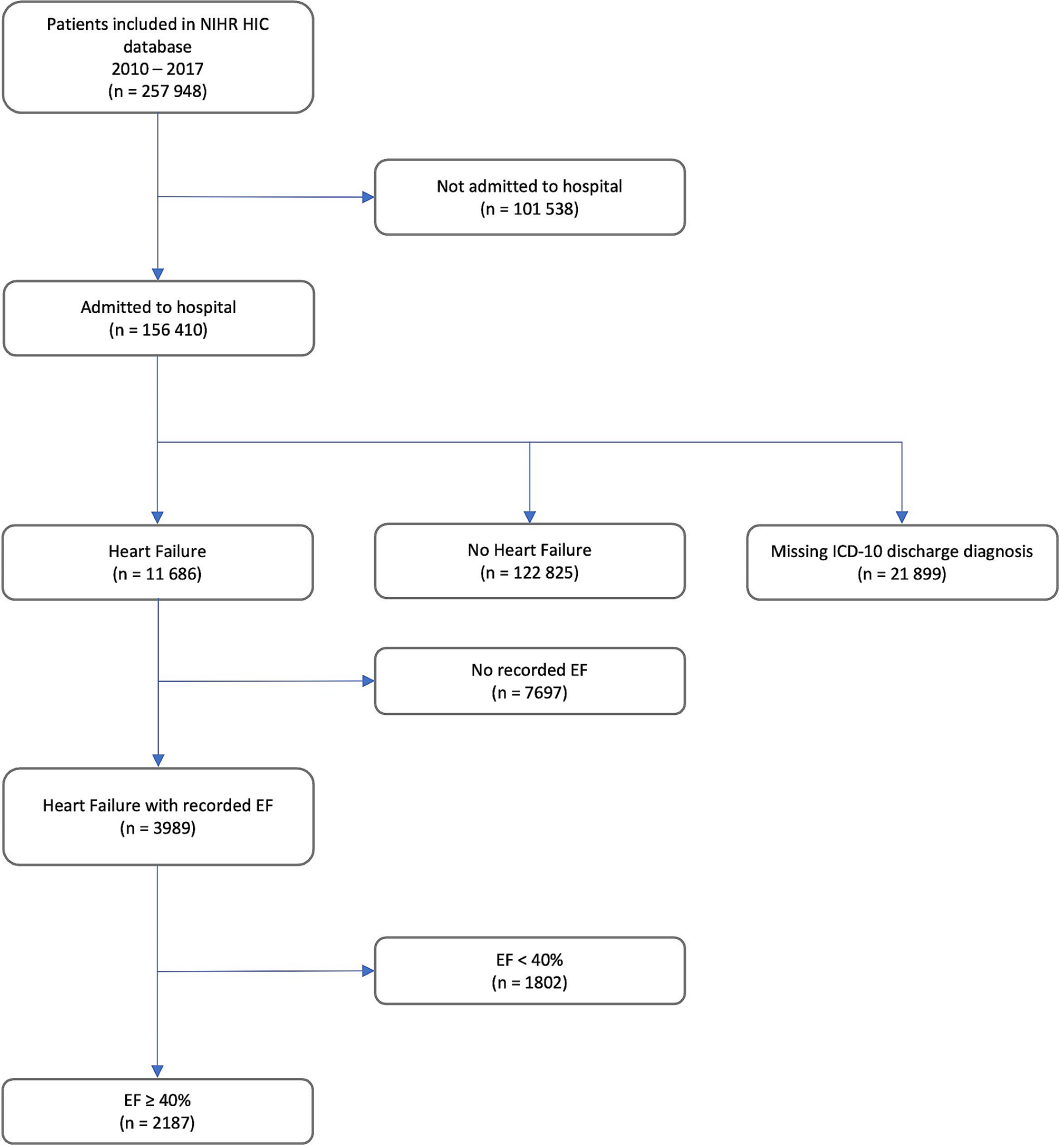
STROBE diagram. EF indicates ejection fraction; HIC, Health Informatics Collaborative; ICD-10, International Classification of Diseases 10th Revision; NIHR, National Institute for Health Research; STROBE, Strengthening the Reporting of Observational Studies in Epidemiology

Of 3,989 patients with a diagnosis of HF and a recorded LVEF, 2,187 (54.8%) had a LVEF *≥* 40% and were included in the study (Fig. 1). Mean age was 74 ± 14.5 years and 1,202 (55%) were female. Median LVEF was 54% (interquartile range 45–60%).

### Cluster analysis

**Fig. 2:**
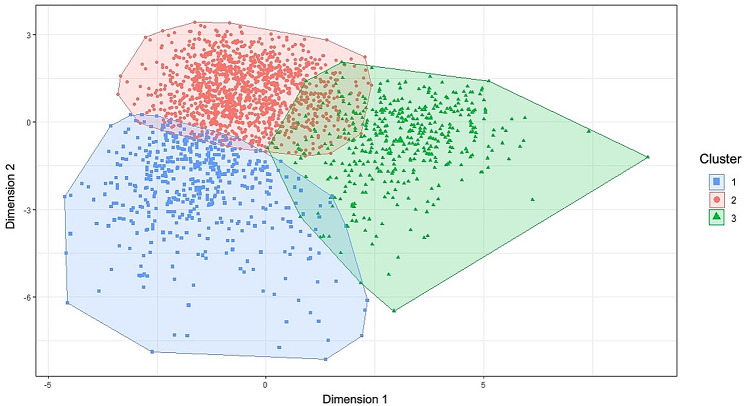
Cluster plot illustrating the graphical representation of clusters from the k-means algorithm. Dimensions represent the principal components explaining the largest variation in data. Dimension 1 and Dimension 2 capture 9.1% and 7.8% of the variance, respectively. Each of the dots represent individual participants

The optimal number of clusters selected for k-means clustering was three (Supplemental Fig. 2), which demonstrated good separation of clusters (Fig. 2), and high cluster stability over bootstrapped samples (mean Jaccard coefficients 0.87, 0.74 and 0.87 for each cluster, respectively). The DBSCAN clustering algorithm was unable to separate the patients into clusters (Supplemental Fig. 3). The model-based algorithm identified four clusters, but there was significant overlap and mean Jaccard coefficients were lower (Supplemental Fig. 4). Therefore, the three clusters identified using k-means clustering were selected for further analysis and hereafter referred to as phenogroups.

### Phenogroup characteristics

Characteristics of the three phenogroups differed significantly (Table 1). Phenogroup 1 comprised younger, predominantly female (71.7%) patients, with high rates of cardiometabolic conditions, such as hypertension, hypercholesterolaemia and obesity, and severe coronary artery disease (CAD) (87.9%). This phenogroup had the lowest LVEF (47% (43–55%); compared to 55% (48–61%) and 55% (45–60%) in phenogroups 2 and 3 respectively; *p* < 0.001) and markedly higher troponin levels.

**Table 1:** Comparison of clinical characteristics between phenogroups

	Phenogroup 1 (*n* = 438)	Phenogroup 2 (*n* = 1273)	Phenogroup 3 (*n* = 476)	*P* Value
Age (years)	70.7 (12.3)	74.2 (15.4)	75.1 (13.8)	< 0.001
Male	124 (28.3%)	672 (52.8%)	189 (39.7%)	< 0.001
**Cardiometabolic disease**				
Hypertension	317 (72.4%)	656 (51.5%)	314 (66.0%)	< 0.001
Diabetes	162 (37.0%)	296 (23.3%)	266 (55.9%)	< 0.001
Hypercholesterolaemia	193 (44.1%)	297 (23.3%)	104 (21.8%)	< 0.001
Obesity	66 (15.1%)	93 (7.3%)	31 (6.5%)	< 0.001
**Cardiovascular disease**				
Previous myocardial infarction	179 (40.9%)	124 (9.7%)	75 (15.8%)	< 0.001
Acute myocardial infarction	84 (19.2%)	63 (4.9%)	60 (12.6%)	< 0.001
Atrial fibrillation	61 (13.9%)	392 (30.8%)	139 (29.2%)	< 0.001
Ischaemic stroke	7 (1.6%)	39 (3.1%)	9 (1.9%)	< 0.001
Transient ischaemic attack	< 5 (< 2%)	< 5 (< 1%)	6 (1.3%)	0.035
Peripheral vascular disease	29 (6.6%)	53 (4.2%)	58 (12.2%)	< 0.001
Hypertrophic cardiomyopathy	< 5 (< 2%)	10 (0.8%)	< 5 (< 2%)	0.437
**Renal disease**				
Chronic kidney disease	28 (6.4%)	51 (4.0%)	236 (49.6%)	< 0.001
Acute kidney injury	38 (8.7%)	89 (7.0%)	238 (50.0%)	< 0.001
Amyloidosis	< 5 (< 2%)	< 5 (< 1%)	< 5 (< 2%)	0.237
**Respiratory disease**				
COPD	43 (9.8%)	230 (18.1%)	75 (15.8%)	< 0.001
Asthma	26 (5.9%)	98 (7.7%)	16 (3.4%)	0.004
Interstitial lung disease	< 5 (0.2%)	26 (2.0%)	< 5 (0.8%)	0.007
Pneumonia	56 (12.8%)	222 (17.4%)	149 (31.3%)	< 0.001
**Frailty**				
Dementia	< 5 (< 2%)	44 (3.5%)	13 (2.7%)	0.004
History of falls	< 5 (< 2%)	77 (6.0%)	23 (4.8%)	< 0.001
Osteoporosis	7 (1.6%)	82 (6.4%)	18 (3.8%)	< 0.001
Need for home assistance	< 5 (< 2%)	< 5 (< 1%)	< 5 (< 2%)	0.491
**Laboratory investigations**				
Haemoglobin (g/dl)	12.9 (2.15)	12.2 (2.17)	10.3 (2.1)	< 0.001
White cell count (10*9/l)	10.4 (7.9–13.3)	8.6 (6.5–11)	9.4 (6.9–12.8)	< 0.001
Platelet count (10*9/l)	228 (177.3–278)	214 (168–279)	199 (147-268.3)	< 0.001
Sodium (mmol/l)	137 (4.54)	138 (4.9)	137 (5.8)	< 0.001
Potassium (mmol/l)	4.32 (0.54)	4.3 (0.6)	4.87 (0.8)	< 0.001
Urea (mmol/l)	7.2 (5.5–9.9)	7.4 (5.7–9.7)	18.3 (13.4–24.6)	< 0.001
Creatinine (umol/l)	87 (73–115)	85 (69–108)	193 (145-309.3)	< 0.001
eGFR (mL/min/1.73m^2^)	51.5 (37.6–74)	51.5 (37–71)	17.9 (11.4–26)	< 0.001
C-reactive protein (mg/l)	9.4 (4.9–40.9)	13.7 (5–45)	37.4 (10-96.3)	< 0.001
Troponin (ratio of assay ULN)	112.7 (6.5-725.4)	2.4 (1-6.9)	6.4 (3-20.3)	< 0.001
**Echocardiography**				
LV ejection fraction (%)	47 (42.9–55)	55 (48–61)	55 (45–60)	< 0.001
LV end diastolic dimension (cm)	4.9 (0.7)	4.7 (0.8)	4.8 (0.8)	< 0.001
LV end systolic dimension (cm)	3.5 (0.7)	3.3 (0.8)	3.3 (0.8)	< 0.001
**Invasive procedures at index admission**				
Coronary angiography	438 (100%)	31 (2.4%)	52 (10.9%)	< 0.001
Severe CAD on angiography	385 (87.9%)	< 5 (< 1%)	43 (9.0%)	< 0.001
PCI	329 (75.1%)	< 5 (< 1%)	23 (4.8%)	< 0.001
CABG	55 (12.6%)	69 (5.4%)	32 (6.7%)	< 0.001

Data are median (IQR), mean (SD) or n (%) as appropriate. Counts < 5 can only be presented as ‘<5’ due to data protection requirements. CABG indicates coronary artery bypass grafting; CAD, coronary artery disease; COPD, chronic obstructive pulmonary disease; eGFR, estimated glomerular filtration rate; LV, left ventricular; PCI, percutaneous coronary intervention; ULN, upper limit of normal

Phenogroup 2 comprised comparatively more males (52.8%), and patients were generally more frail, displaying relatively higher rates of dementia, falls, osteoporosis and chronic lung disease. Atrial fibrillation was also common (30.8%).

Over half the patients in phenogroup 3 were diabetic (55.9%, compared to 37% and 23.3% in phenogroups 1 and 2 respectively; *p* < 0.001). Renal function was considerably worse (eGFR 17.9 (11.4–26); compared to 51.5 (37.6–74) and 51.5 (37–71) respectively; *p* < 0.001), and c-reactive protein was higher (37.4 (10–96.3); compared to 9.4 (4.9–40.9) and 13.7 (5–45) respectively; *p* < 0.001).

### Phenogroup outcomes

**Fig. 3:**
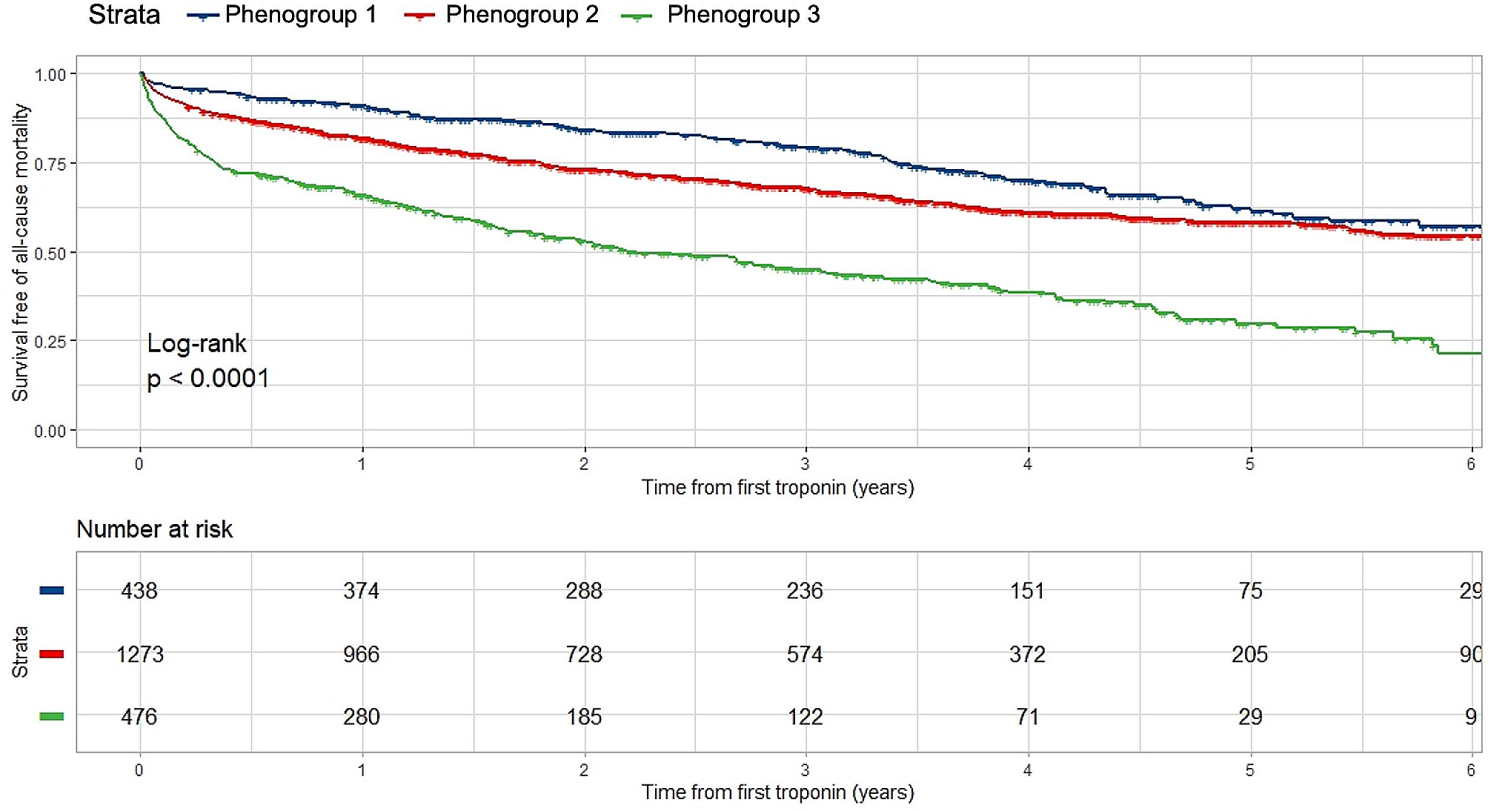
Kaplan-Meier curves for all-cause mortality. Survival free of all-cause mortality stratified by phenogroup

During a median follow-up of 2.4 (IQR: 1.0–4.1) years, the primary outcome of all-cause mortality occurred in 842 (38.5%) patients and the secondary outcome of HHF occurred in 518 (23.6%) patients. As demonstrated in Table 2; Fig. 3, phenogroups displayed distinct survival profiles, with a stepwise increasing risk of all-cause mortality from phenogroup 1 to 3 (*p* < 0.001). Addition of the phenogroup variable to a series of nested Cox proportional hazard models significantly improved the performance of each model (Table 3). Phenogroups were not predictive of HHF (Supplemental Fig. 5, Table 2).

**Table 2:** Association of phenogroups with outcome

	Phenogroup 1 (*n* = 438)	Phenogroup 2 (*n* = 1273)		Phenogroup 3 (*n* = 476)	
	HR (95% CI)	HR (95% CI)	*P* Value	HR (95% CI)	*P* Value
All-cause mortality	1	1.38 (1.13–1.69)	**0.002**	2.99 (2.41–3.70)	**< 0.001**
Hospitalisation for heart failure	1	1.10 (0.88–1.37)	0.420	0.97 (0.73–1.28)	0.810

Unadjusted Cox proportional hazards model used to evaluate association between phenogroups and all-cause mortality. Fine-Gray competing risks model used to evaluate association between phenogroups and hospitalisation for heart failure

**Table 3:** Added prognostic value of phenogroups

Model	C-statistic (SE)	C-statistic (SE) with addition of phenogroup	*P*-value for addition of phenogroup (LLR test)
Age, Sex	0.597 (0.010)	0.647 (0.010)	< 0.001
Age, Sex, Diabetes, Hypertension, Obesity	0.601 (0.010)	0.651 (0.010)	< 0.001
Age, Sex, Diabetes, Hypertension, Obesity, COPD	0.617 (0.010)	0.662 (0.010)	< 0.001
Age, Sex, Diabetes, Hypertension, Obesity, COPD, eGFR, LVEF	0.638 (0.010)	0.664 (0.010)	< 0.001
Age, Sex, Diabetes, Hypertension, Obesity, COPD, eGFR, LVEF, Troponin*, Haemoglobin, Severe CAD	0.671 (0.009)	0.683 (0.009)	< 0.001
Age, Sex, Diabetes, Hypertension, Obesity, COPD, eGFR, LVEF, Troponin*, Haemoglobin, Severe CAD, Dementia, Atrial fibrillation, Sodium, C-reactive protein	0.691 (0.009)	0.700 (0.009)	< 0.001

A series of nested Cox proportional hazard models for the primary outcome. Added prognostic value of phenogroups evaluated by examining C-statistic for both base model and base model with addition of phenogroup variable. In addition, a log-likelihood ratio test for addition of phenogroup to base model was performed. CAD indicates coronary artery disease; COPD, chronic obstructive pulmonary disease; eGFR, estimated glomerular filtration rate; LLR, log-likelihood ratio test; LVEF, left ventricular ejection fraction; SE, standard error. *The troponin variable is a log-transformed value of the troponin and assay upper limit of normal ratio

## Discussion

The principal findings of this study are that unsupervised machine learning techniques applied to routinely collected electronic health record data were able to identify phenogroups with distinct clinical characteristics and unique survival profiles.

HF with preserved or mildly reduced ejection fraction includes a heterogenous group of patients with multiple pathophysiological mechanisms. Previous attempts at reclassification with machine learning cluster analyses have been limited by small cohorts or clinical trial data [7–10, 22–24]. The current study is the first to utilise routinely collected electronic health record data from a large cohort for this purpose. Electronic health record data enables efficient generation of large, often unselected cohorts that are reflective of clinical practice, both in terms of patient characteristics and available data, and thus offer potential advantages over data from clinical trials and specialist centre registries. As more hospitals and healthcare centres establish contemporary electronic health records, and data curation and extraction methods become more effective, the utility of routinely collected health data for research is set to grow.

The NIHR Health Informatics Collaborative includes electronic health records for more than 250,000 patients, which enabled a relatively large cohort of patients with HF with preserved or mildly reduced ejection fraction to be included in the current study. An LVEF threshold of 40% was used in keeping with many previous and most ongoing studies [5, 6, 25].

Three distinct phenogroups were identified. Phenogroup 1 comprised younger, predominantly female patients with high rates of cardiometabolic conditions and CAD, the lowest LVEF and the lowest risk of death. Phenogroup 2 comprised comparatively more males and was characterised by markers of frailty. Phenogroup 3 displayed evidence of systemic inflammation, with high rates of diabetes and renal dysfunction, and the highest risk of death.

A subgroup similar to phenogroup 3 has been identified in previous machine learning-based studies. Latent-class analysis of clinical, circulating biomarker and imaging data from the Treatment of Preserved Cardiac Function Heart Failure with an Aldosterone Antagonist Trial (TOPCAT) [10], and unsupervised cluster analysis of 363 circulating proteins in a cohort of 429 patients with heart failure with preserved ejection fraction (HFpEF) [9], both identified a subgroup characterised by a high prevalence of diabetes and renal dysfunction, elevated inflammatory markers, and a high rate of a composite outcome of HHF or all-cause mortality. Co-morbidity driven systemic inflammation leading to microvascular endothelial dysfunction is a commonly hypothesised HFpEF disease mechanism [26], and it may be that it represents an important pathophysiological process in this subgroup. Whilst causal investigation is required, it is noteworthy that different machine learning techniques applied to differing modes of data from a range of study types have consistently identified a similar phenogroup, suggesting that endotypes do indeed exist and reclassification of is feasible and clinically meaningful.

In studies by Woolley et al. [9] and Kao et al. [22], subgroups with the highest prevalence of CAD, and highest troponin levels where available, were at high risk of adverse outcomes (composites of HHF or all-cause mortality, and cardiovascular hospitalisation or all-cause mortality, respectively). Conversely, in the current study, whilst almost 90% of patients in phenogroup 1 had evidence of severe CAD on invasive angiography and troponin levels were substantially higher, phenogroup 1 was associated with the lowest risk of death. In the studies by Woolley et al. and and Kao et al., other markers of adverse outcome (particularly death) clustered with CAD, such as older age, male, chronic obstructive pulmonary disease and anaemia, whereas in the current study CAD clustered with younger, predominantly female patients. All patients in phenogroup 1 underwent invasive coronary angiography and almost 90% underwent revascularisation, which may have influenced the cluster analysis; as well as severity of CAD, the decision to undertake revascularisation summates multiple patient factors that may not be well recorded.

Such differences also serve to illustrate other key factors in this kind of analysis. First, the nature of the cohort strongly influences the nature of the subgroups identified. One of the strengths of the current cohort is the relatively high proportion of patients with invasive angiogram-documented CAD severity, but a potential limitation is that inclusion in the NIHR Health Informatics Collaborative database, from which the current cohort was identified, requires patients to have had circulating troponin measured, which could skew towards an ischaemic population and limit the transferability of these findings to other HFpEF cohorts in which troponin is not routinely measured. Nonetheless, the proportion of patients with a history of previous myocardial infarction (17%) is in keeping with other HFpEF studies e.g., 29% in the EMPEROR-Preserved trial [5]. Similarly, the cohort included in the study by Woolley et al. [9] was derived from a wider cohort of patients with HF who were considered to be on suboptimal medical treatment [27], and the studies by Kao et al. [22] and Cohen et al. [10] derived patients from clinical trials.

Second, the prognostic utility of identified phenogroups depends on the choice of outcome measure. All-cause [9, 22] and cardiovascular [7, 10] mortality have both been used in prior HF machine learning analyses. All-cause mortality was used in the current study because it is more relevant for patients, it is more accurate, more than half of deaths in HFpEF are non-cardiovascular [5], and cause of death data were not available. In most previous studies, the measure of mortality is included alongside a measure of morbidity, such as HHF or cardiovascular hospitalisation, in a composite outcome. This is typically done to increase study power and was not necessary here due to the large number of deaths. Indeed, HHF is a markedly different outcome to death, highlighted in this study by the similar rates of HHF despite significantly different between-phenogroup mortality. Whilst this may be explained by the availability of only HHF episodes occurring at the hospital of index admission, it is in keeping with most such studies. Understanding the relationship between phenogroups and each outcome separately may, for example, facilitate interventions aimed at preventing HHF. It is also important to recognise that, rather than for predicting outcome, the main reasons for identifying distinct subgroups are ultimately to identify underlying causal mechanisms, identify and prioritise therapeutic targets, and discover diagnostic biomarkers specific to each; it may be that different subgroups have similar outcome rates.

Finally, the choice of clustering algorithm can have a significant impact on the type and number of derived subgroups. Whilst the clustering algorithms in this study were not quantitatively compared, k-means clustering was selected for further analysis as it derived the most stable clusters.

A limitation of the study is the relatively sparse patient characterisation, which predominantly comprised categorical variables and included minimal laboratory and echocardiographic data and, therefore, limited the reliability of the HFpEF diagnosis. Additionally, variables were selected according to clinical practice prior to clustering, which, while similar to previous studies, may limit the ability of the unsupervised machine learning algorithms to identify novel clusters. External validation was also not performed. Future studies should aim to perform externally validated cluster analyses on highly characterised, multimodal data, including deep clinical phenotyping, imaging, multi-omics and electronic health record data, at scale. The United Kingdom HFpEF Registry (UK HFpEF; NCT05441839 https://www.ukhfpef.org/) is a UK national initiative that aims to do just that. Other limitations are described earlier.

## Conclusions

Through applying unsupervised machine learning techniques to routinely collected electronic health record data, this study identified phenogroups with distinct clinical characteristics and unique survival profiles.

### Electronic supplementary material

Below is the link to the electronic supplementary material.

Supplementary Material 1

## Data Availability

The datasets generated and/or analysed during the current study are not publicly available due to ethical restrictions but are available from the corresponding author on reasonable request.

## Abbreviations

DBSCAN: Density–based spatial clustering and application with NOISE
HF: Heart failure
HFpEF: Heart failure with preserved ejection fraction
HHF: Hospitalisation for heart failure
ICD: 10–International Classification of Diseases 10th Revision
LVEF: Left ventricular ejection fraction
NIHR: National Institute for Health Research
NICOR: National Institute for Cardiovascular Outcomes Research
