# Supplementary material for: Phenogrouping heart failure with preserved or mildly reduced ejection fraction using electronic health record data
Source: BMC Cardiovasc Disord. 2024 Jul 5;24:343. doi: 10.1186/s12872-024-03987-9 (PMC11229019; doi:10.1186/s12872-024-03987-9)
Supplement: Supplementary file 1 — Supplementary Material 1 [file 12872_2024_3987_MOESM1_ESM.pdf]

Phenogrouping heart failure with preserved ejection fraction using electronic health record data

Supplemental file

Supplemental methods

Variable selection and data pre-processing

47 variables from the National Institute for Health Research (NIHR) Health Informatics Collaborative database were considered as candidates for cluster analysis. Clinical variables, such as cardiovascular risk factors, cardiovascular disease, renal disease, respiratory disease, and frailty, were extracted from the database using International Classification of Diseases 10th Revision (ICD-10) discharge codes. Outcome data were excluded. Variables with $\geq$ 20% missing data were excluded (haemoglobin A1C, total cholesterol, high-density lipoprotein cholesterol, low-density lipoprotein cholesterol, serum triglycerides, serum glucose), leaving a total of 42 variables (Supplemental Table 2). The troponin variable represented the peak troponin value on the patients' index admission. As the test was performed using many different assays, the results were standardised by transforming each result to a ratio of the troponin value divided by the troponin assay upper limit of normal (ULN). 424 patients (19%) had at least one missing value. Missing data (summarised in Supplemental Figure 1) were imputed using the *missForest* package in R, which is an iterative imputation method based on a random forest, that constitutes a multiple imputation scheme by averaging over multiple regression trees (1). The out of bag error was 0.07. Following imputation, categorical variables (all binary) were transformed to numerical, and all variables were scaled and standardised to a mean of 0 and standard deviation of 1.

Clustering methodology

Three clustering methods were applied:

Density-based clustering

Density-Based Spatial Clustering and Application with Noise (DBSCAN), a type of density-based clustering algorithm, was applied to the data initially. DBSCAN has several advantages. In contrast to other clustering methods, it does not require the user to specify the number of clusters. Furthermore, DBSCAN is more suited to finding arbitrary shaped clusters and to detect outliers in data (2). The algorithm requires the input of two parameters: the epsilon value, which is a distance metric around a data point 'x'; and the minimum points value, which describes the minimum number of other data points, or neighbours, within the radius of the epsilon.

The dbscan function in the fpc R package was applied. The minimum points value was calculated by multiplying the dimensionality of the data by 2, creating a minimum points value of 84. The optimal epsilon was then selected by plotting a k-distance plot (k corresponds to the minimum points value) and choosing the value at the 'elbow' point (3).

Model-based clustering

Gaussian mixture modelling, as implemented by Shah et al using the mclust package in R, is a form of model-based clustering that achieves parameter estimation with the use of an expectation-maximisation algorithm (4, 5). A variety of covariance structures can be explored, and the optimal number of clusters can be determined with maximisation of the Bayesian Information Criterion (BIC). The BIC penalises model complexity and allows selection of models with overall better fit. Between 1 and 9 clusters were explored.

Partition-based clustering

K-means clustering, implemented here using the *stats* package in R, is a commonly used partition-based clustering approach that divides the data into k-number of clusters (6). The number of clusters

(k) is pre-defined by the user and the algorithm initiates with a random selection of k observations which act as cluster centroids. The remaining observations are then assigned to the closest centroid, calculated using the Euclidean distance. Following this, the centroids of each cluster are adjusted, and the observations are reassigned using the updated centroid values. Finally, the whole process is iteratively repeated to minimise within-cluster variation (defined as the sum of squared Euclidean distances) and achieve convergence.

Several methods, such as the average silhouette or gap statistic, exist to select the optimal number of clusters. In this study, k was determined using the NbClust package in R. NbClust selects the optimal cluster number by choosing the number that is calculated by the majority of 30 different indices (Supplemental Figure 2) (7). The algorithm was limited to select between 3 and 8 clusters. The optimal number chosen by most indices was 3 clusters.

Cluster stability

To investigate the validity of the clusters, cluster stability was assessed by calculating the mean Jaccard coefficient for each cluster across bootstrapped replicates (8). The Jaccard coefficient acts as a similarity measure, comparing the similarity of the clusters when the algorithm is repeated over bootstrapped samples. Values range between 0 to 1, with a value closer to 1 suggesting a greater degree of similarity and stability.

Cluster stability was evaluated using the clusterboot package in R. Given that the DBSCAN algorithm was unable to converge the data beyond a single cluster, cluster stability was not assessed. The model-based clustering and k-means clustering approaches were repeated 100 and 1000 times respectively (1000 replicates was deemed too computationally inefficient for the model-based clustering approach).

Supplemental Table 1: National Institute for Cardiovascular Outcomes Research (NICOR) definition of heart failure (implemented in the National Heart Failure Audit in England and Wales)

ICD-10 code	Definition
I11.0	Hypertensive heart disease with (congestive) heart failure
I25.5	Ischaemic cardiomyopathy
I42.0	Dilated cardiomyopathy
I42.9	Cardiomyopathy, unspecified
I50.0	Congestive heart failure
I50.1	Left ventricular failure
I50.9	Heart failure, unspecified

ICD-10 indicates International Classification of Diseases, 10th Revision

Supplemental Table 2: Variables used in cluster analysis

	Demographics	Clinical	Laboratory	Echocardiography	Invasive procedures at index admission
**Categorical Variables**	Sex	**Cardiovascular risk factors**<br>Hypertension<br>Diabetes<br>Hypercholesterolaemia<br>Obesity<br>Previous myocardial infarction<br>**Cardiovascular disease**<br>Atrial fibrillation<br>NSTEMI<br>Ischaemic stroke<br>Peripheral vascular disease<br>Hypertrophic cardiomyopathy<br>**Renal disease**<br>Chronic kidney disease<br>Acute kidney injury<br>Amyloidosis<br>**Respiratory disease**<br>COPD<br>Asthma<br>Interstitial lung disease<br>Pneumonia<br>**Frailty**<br>Dementia<br>History of falls<br>Osteoporosis<br>Need for home assistance<br>**Admission type**<br>Elective			Coronary angiography<br>Severe CAD on angiography<br>PCI<br>CABG
**Continuous Variables**	Age		**Full blood count**<br>Haemoglobin<br>White cell count<br>Platelet count<br>**Renal profile**<br>Sodium<br>Potassium<br>Urea<br>Creatinine<br>eGFR<br>**Miscellaneous**<br>C-reactive protein<br>Troponin (ratio of assay ULN)	LV ejection fraction<br>LV end diastolic dimension<br>LV end systolic dimension	

CABG indicates coronary artery bypass grafting; CAD, coronary artery disease; COPD, chronic obstructive pulmonary disease; eGFR, estimated glomerular filtration rate; LV, left ventricular; NSTEMI, non-ST elevation myocardial infarction; PCI, percutaneous coronary intervention; ULN, upper limit of normal.

Supplemental Figure 1: Frequency of missing data

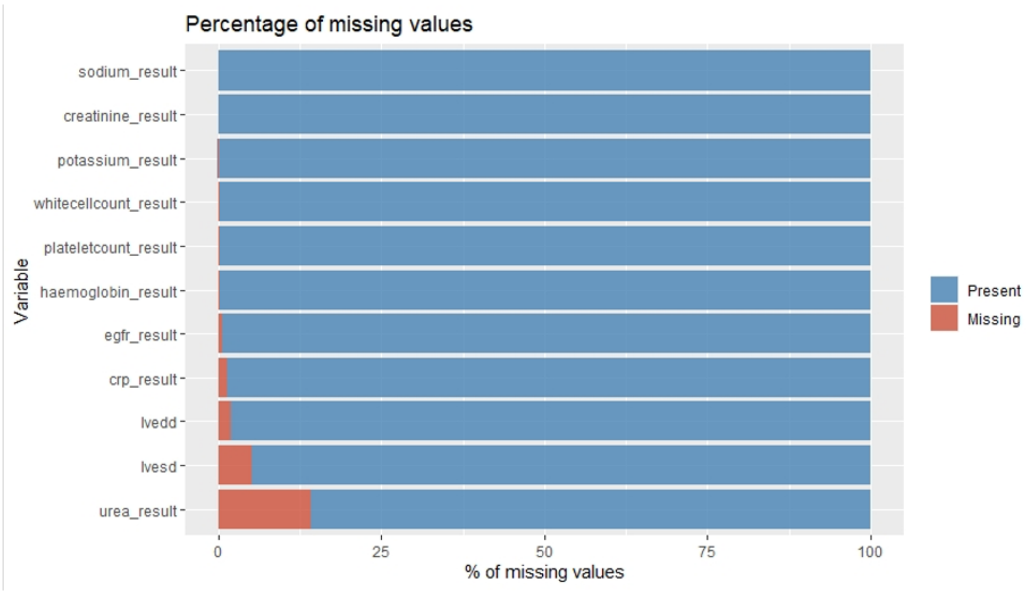

crp indicates c-reactive protein; egfr, estimated glomerular filtration rate; lvedd, left ventricular end diastolic dimension; lvesd, left ventricular end systolic dimension

Supplemental Figure 2: Optimal number of clusters using *NbClust* package

NbClust determines the optimal number of clusters (k) using 30 indices. The algorithm was limited to between 3 to 8 clusters, with k = 3 selected most frequently.

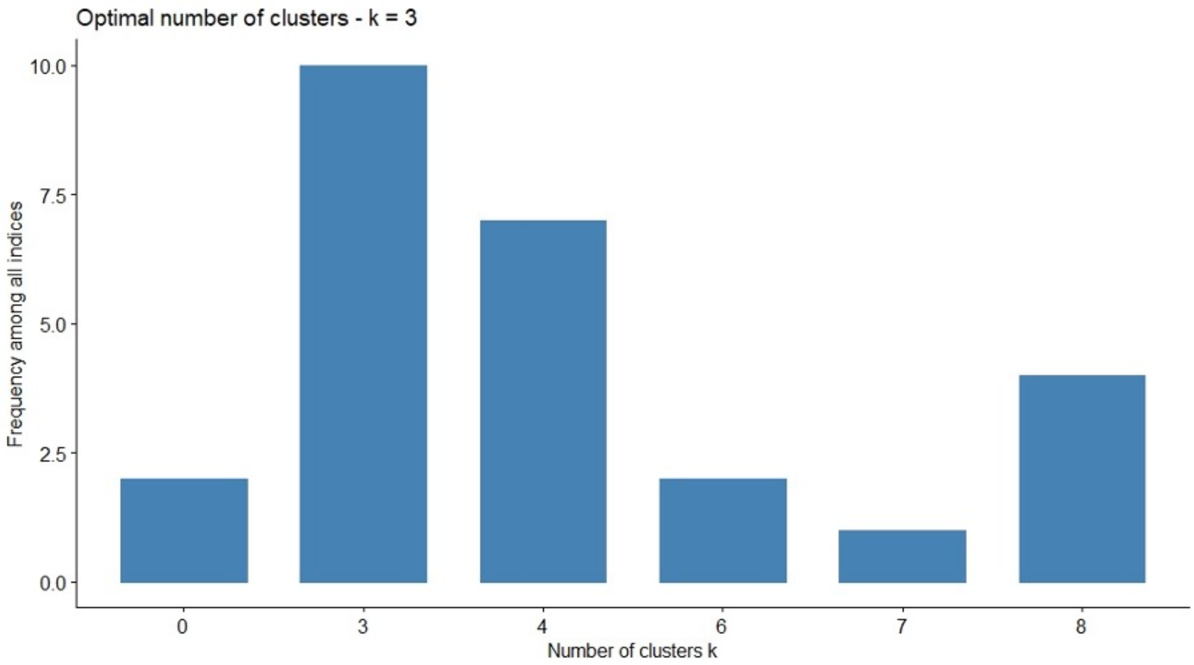

Supplemental Figure 3: Cluster plot of data created using DBSCAN.

Dimensions represent the principal components explaining the largest variation in data. Each of the dots represent individual participants.

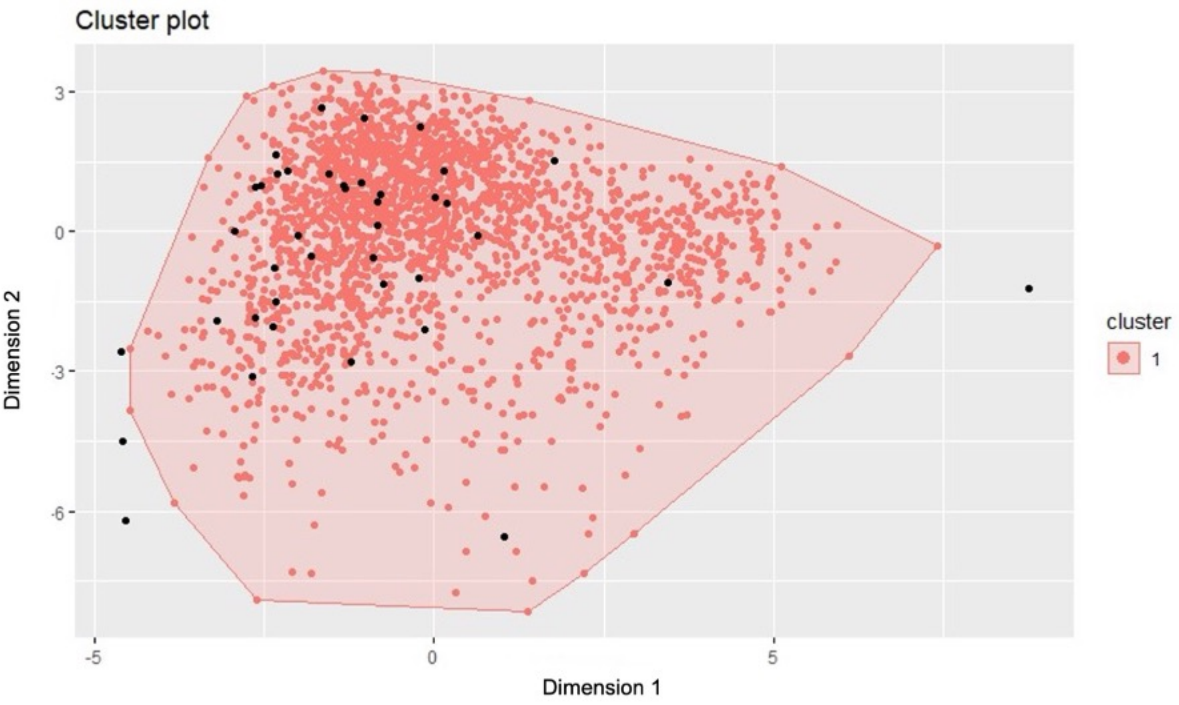

Supplemental Figure 4: Model-based clustering using *mclust* package.

Cluster plot. Dimensions represent the principal components explaining the largest variation in data. Each of the dots represent individual participants. Mean cluster Jaccard coefficients 0.62, 0.63, 0.78 and 0.92.

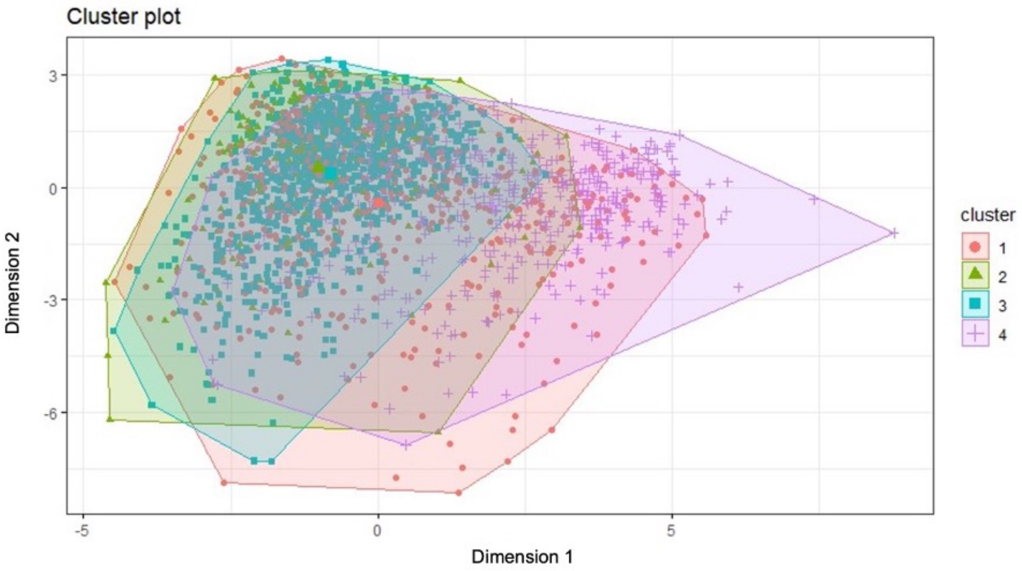

Supplemental Figure 5: Kaplan-Meier curves for further hospitalisation for heart failure

Survival free of further hospitalisation for heart failure stratified by phenogroup. No significant difference between phenogroups.

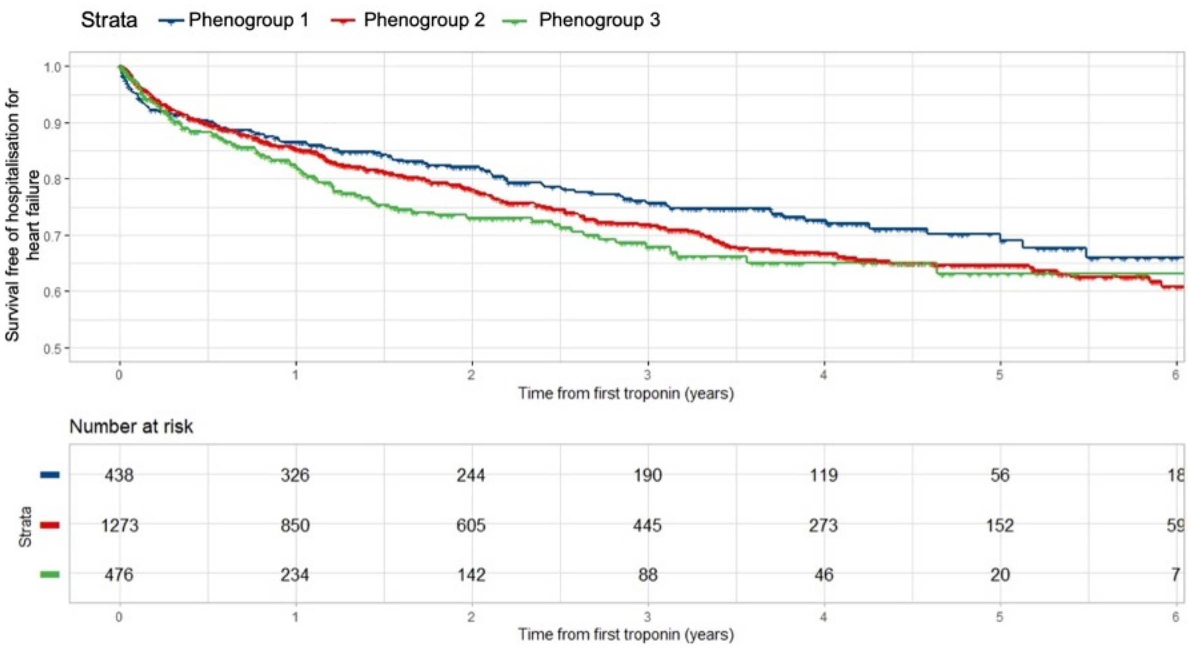

References

1. Stekhoven DJ, Bühlmann P. MissForest—non-parametric missing value imputation for mixed-type data. Bioinformatics. 2011;28(1):112-8.

2. Ester M, Kriegel HP, Sander J, Xiaowei X. A density-based algorithm for discovering clusters in large spatial databases with noise. Conference: 2 international conference on knowledge discovery and data mining, Portland, OR (United States), 2-4 Aug 1996; Other Information: PBD: 1996; Related Information: Is Part Of Proceedings of the second international conference on knowledge discovery & data mining; Simoudis, E; Han, J; Fayyad, U [eds]; PB: 405 p; United States: AAAI Press, Menlo Park, CA (United States); 1996. p. Medium: X; Size: pp. 226-31.

3. Sander J, Ester M, Kriegel H-P, Xu X. Density-Based Clustering in Spatial Databases: The Algorithm GDBSCAN and Its Applications. Data Mining and Knowledge Discovery. 1998;2(2):169-94.

4. Shah SJ, Katz DH, Selvaraj S, Burke MA, Yancy CW, Gheorghiade M, et al. Phenomapping for Novel Classification of Heart Failure With Preserved Ejection Fraction. Circulation. 2015;131(3):269-79.

5. Fraley C, Raftery AE. Model-Based Clustering, Discriminant Analysis, and Density Estimation. Journal of the American Statistical Association. 2002;97(458):611-31.

6. Hartigan JA, Wong MA. Algorithm AS 136: A K-Means Clustering Algorithm. Journal of the Royal Statistical Society Series C (Applied Statistics). 1979;28(1):100-8.

7. Charrad M, Ghazzali N, Boiteau V, Niknafs A. NbClust: An R Package for Determining the Relevant Number of Clusters in a Data Set. Journal of Statistical Software. 2014;61(6):1 - 36.

8. Hennig C. Cluster-wise assessment of cluster stability. Computational Statistics & Data Analysis. 2007;52(1):258-71.
